# Environmental DNA Based Assessment of Fish Diversity in the Yarlung Zangbo River, Tibetan Plateau

**DOI:** 10.1002/ece3.72496

**Published:** 2025-11-10

**Authors:** Haiyu Wang, Qianqian Wu, Shenhui Li, Hongyu Jin, Zepeng Zhang, Wanqiao Lu, Fei Liu, Guishuang Wang, Lei Li

**Affiliations:** ^1^ Heilongjiang River Fishery Research Institute of Chinese Academy of Fishery Sciences, Heilongjiang River Basin Fishery Resources and Environment Scientific Observation and Experiment Station of the Ministry of Agriculture and Rural Affairs Harbin China; ^2^ College of Fisheries and Life Sciences Shanghai Ocean University Shanghai China; ^3^ Graduate School of Human Development and Environment Kobe University Kobe Hyogo Japan; ^4^ Institute of Aquatic Sciences, Tibet Autonomous Region Academy of Agricultural and Animal Husbandry Sciences Lhasa Tibet China

**Keywords:** alien species, community structure, environmental DNA (eDNA), fish diversity, Yarlung Zangbo River

## Abstract

Biodiversity is under unprecedented threat globally, with ecosystems vulnerable to climate change and detrimental anthropogenic impacts. Accurate assessments of biodiversity are essential to the development of effective conservation strategies. We investigated the fish diversity of the Yarlung Zangbo River of the Tibetan Plateau using environmental DNA (eDNA) technology. Water samples collected from 18 sampling sites revealed 18 fish species, including six unique to Tibet and one on China's list of State Key Protected Wild Animals, *Oxygymnocypris stewartii* . *Schizopygopsis younghusbandi* is widely distributed, with stable populations in all sampling sites. Non‐metric multidimensional scaling analyses showed fish community composition to vary with elevation, although the data did not reach significance. Spearman correlation analyses revealed significant associations of fish species with environmental factors including flow velocity, water temperature, conductivity, and total dissolved solids. Eight exotic fish species were detected, highlighting the potential threat posed by non‐native species to the river fish diversity. This study confirms the efficacy of eDNA technology in assessing aquatic biodiversity and its broad applicability as a versatile tool for fish conservation and management, across diverse aquatic ecosystems. In contrast to traditional methods, eDNA offers a non‐invasive, simpler, and more efficient approach for detecting a broad range of species, including those that are rare or difficult to capture. The findings emphasize the need for future research to integrate traditional survey methods and eDNA technology to comprehensively assess ecosystem biodiversity and develop targeted conservation strategies.

## Graphical abstract

The environmental DNA metabarcoding technology applied to assess fish diversity in the Yarlung Zangbo River on the Tibetan Plateau overcomes the limitations of traditional survey methods in a high elevation habitat. (1) Expanding Fish Diversity Discovery: eDNA analysis yielded the identification of 18 fish species, including 6 Tibetan endemics and the protected *Oxygymnocypris stewartii* , providing valuable new data on the region's fish assemblage. (2) Validating eDNA effectiveness: Demonstrating eDNA's ability to accurately assess fish communities in a high‐altitude river, establishing a reliable tool for future monitoring. (3) Highlighting invasive species threats: Identifying 7 alien species underscores the need for targeted management strategies to protect native biodiversity. (4) Revealing environmental influences: Linking factors like altitude and water temperature to fish community structure, providing insights into the drivers of diversity in this unique ecosystem.

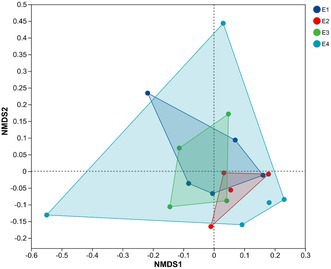

## 1 Introduction

The Yarlung Zangbo River, originating in the Tibetan Plateau, is one of the world's highest rivers (Immerzeel et al. 2010) and the average altitude exceeds 3000 m. It flows eastward through the Himalayan Mountains, forming a U‐shaped canyon landscape characterized by complex topography and a high‐elevation climate that fosters rich biological resources (Wester et al. 2019; Myers et al. 2000). As the longest river in the Tibet Autonomous Region, the Yarlung Zangbo is an important water resource with far‐reaching impacts on the regional economy and cultural heritage. The health and stability of its watershed ecosystems impact water security and the ecological balance of downstream areas and the entire South Asian region (Zhang et al. 2001). The watershed spans a wide range of ecosystem types including alpine grasslands, mountain forests, and river valley wetlands and provides habitat and breeding places for a multitude of aquatic organisms, among which fish play a key role in energy flow, nutrient cycling, and maintenance of ecosystem stability (Nelson et al. 2016).

Fish diversity is an important indicator of the health of aquatic ecosystems, and its alteration may reflect environmental change and the impact of human activity (Dudgeon et al. 2006). Preliminary studies show the Yarlung Zangbo River basin to be home to a wide range of fishes of the subfamily *Schizothoracinae*, which are adapted to the alpine environment, as well as those of the families Carpophagidae and *Loachophagidae*. Many of the species are unique to the region, making the basin an ideal site for the study of biogeography and evolutionary biology (Zhang et al. 2016). Twenty‐four fish species have been recorded in the basin, with indigenous species contributing the majority. Exotic fish species are found in the middle and lower reaches, posing a potential threat to the survival of the native species. There are significant gaps in knowledge of fish diversity in the Yarlung Zangbo River Basin, especially with respect to the remote high‐elevation areas of the middle and upper reaches. Traditional survey methods (netting and electrofishing) are inefficient and challenging when covering broad areas and have the potential to create irreversible disturbance to fragile ecosystems (Eble et al. 2020). There is an urgent need for technology that can overcome these limitations and facilitate rapid, non‐invasive, and high‐resolution monitoring of fish diversity to provide a scientific basis for the protection of the region's ecology as well as basic data to support biodiversity conservation in the context of climate change (Deiner et al. 2017).

In recent years, the development of environmental DNA (eDNA) technology has provided a sensitive, non‐invasive, and efficient means of monitoring aquatic biodiversity (Thomsen and Willerslev 2015). Environmental DNA metabarcoding based on universal primers enables rapid, accurate, and comprehensive analysis of aquatic communities. It can identify the presence of multiple species simultaneously and be used to analyze species abundance and community composition by designing universal primers for conserved gene fragments (e.g., mitochondrial genes) in specific groups of organisms for PCR amplification and high‐throughput sequencing. Previous research has demonstrated that eDNA metabarcoding is effective in detecting the presence of lake fish species and is highly consistent with the results of traditional fishery surveys (Deiner et al. 2015). Building on these findings, eDNA metabarcoding has been widely used to identify endangered and invasive fish species, demonstrating its considerable potential for species conservation (Thomsen et al. 2012). Compared with traditional survey methods, eDNA technology shows superior potential for biodiversity studies in complex habitats such as alpine rivers. We used eDNA metabarcoding to provide key information on fish species diversity in the Yarlung Zangbo River Basin.

We hypothesized that eDNA analysis would reveal fish community composition and distribution patterns, including invasive species, and identify environmental factors affecting fish community structure. The results of this study are expected to provide a precise ecological baseline for the conservation and management of fish resources in the Yarlung Zangbo River. Specifically, this research will: (1) characterize the fish community composition and distribution patterns using eDNA metabarcoding; (2) identify key environmental factors (e.g., elevation, water temperature, flow velocity) significantly correlated with fish species presence and community structure; and (3) assess the occurrence and distribution of non‐native fish species to inform future monitoring and management efforts. These findings will also establish baseline data for assessing the impacts of climate change and human activity on alpine ecosystems and contribute actionable knowledge for the development of global biodiversity conservation strategies.

## 2 Materials and Methods

### 2.1 Sample Sites and Collection: Characterizing Habitats and Environmental Settings

**FIGURE 1:**
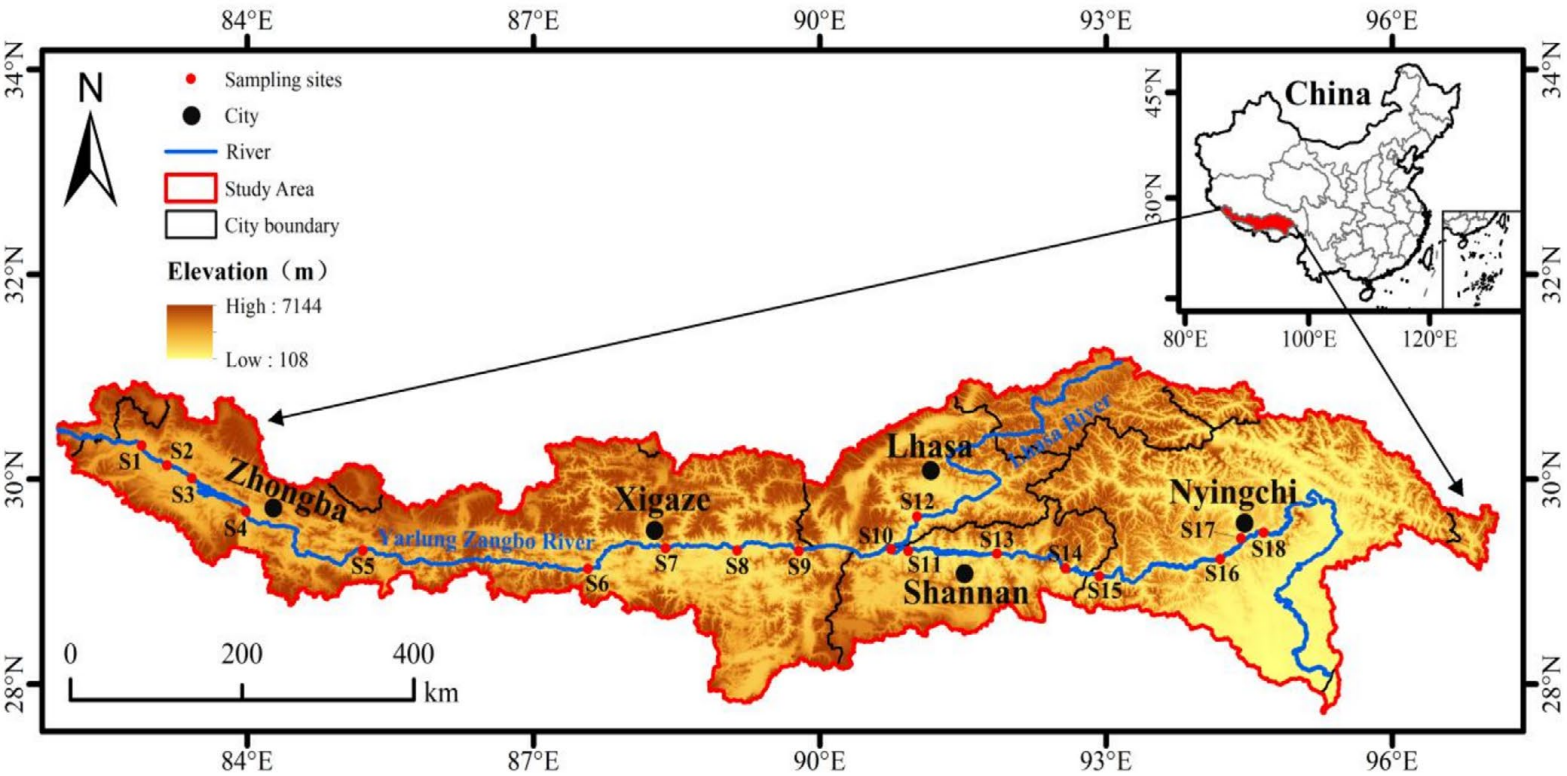
Distribution of sampling sites in the Yarlung Zangbo River (S1–S18), Southwest China, overlaid on a Digital Elevation Model representing elevation above sea level.

Guided by geographic features, hydrological conditions, and preliminary survey data of the Yarlung Zangbo River Basin, sampling sites S1–S18 were selected in November 2024 (Figure 1). These sites cover most of the river at representative elevations and water flow rates, encompassing diverse habitat types including alpine meadows, rocky rapids, and river valley wetlands. The sampling sites were distributed along the river as follows: the upper reaches (S1, S2, S3, S4, S5), the middle reaches (S6, S7, S8, S9, S12), and the lower reaches (S13, S14, S15, S16, S17, S18). The sampling sites located in the upper and middle reaches of the Yarlung Zangbo River, and upstream altitude exceeds 4500 m, and the altitude in the middle reaches exceeds 2800 m. Water samples were collected at a depth of 0.5 m using a multi‐bottle water sampler at each site. Three replicate samples (2 L each) were taken within a 5‐m radius and pooled to form a 6 L composite sample for eDNA analysis. The three replicate samples from each site were then combined to form a composite 6 L sample for eDNA analysis. Samples were held at 4°C in darkness for transport to the laboratory. Within 24 h, samples were filtered through GF/F filters (0.7 μm pore size, 150‐mm diameter) using vacuum filtration. The filter membranes were wrapped in clean aluminum foil, placed in centrifuge tubes, and stored in liquid nitrogen.

### 2.2 Total DNA Extraction From Water Samples

DNA was extracted from the filtered membranes of the collected water samples using the E.Z.N.A. Soil DNA Kit (Omega Bio‐tek, Norcross, GA, USA) according to the manufacturer's protocol. Briefly, the filter membranes were processed with the lysis buffer at 56°C for 30 min to ensure complete cell lysis. Following lysis, DNA was bound to the silica‐based membrane columns, washed with wash buffers to remove contaminants, and finally eluted with 50 μL of nuclease‐free water. The quality of the extracted genomic DNA was then assayed by agarose gel electrophoresis using 1% agarose according to the kit's instructions, and DNA concentration and purity were determined using NanoDrop2000 (Thermo Scientific, USA).

### 2.3 Amplification of Target Gene Fragments

The extracted DNA was used as a template for PCR amplification of 12S rRNA using the forward primer MiFish‐U‐F (GTCGGTAAAACTCGTGCCAGC) and the reverse primer MiFish‐U‐R (CATAGTGGGGGTATCTAATCCCAGTTTG) carrying barcode sequences (Miya et al. 2015). The PCR reaction system consisted of 10 μL 2 × NuHi HiFi MasterMix buffer (TransGen, China), 0.8 μL forward primer (5 μM), 0.8 μL reverse primer (5 μM), and 10 ng template DNA, supplemented with double‐distilled water to 20 μL. The amplification procedure consisted of pre‐denaturation at 94°C for 5 min, 35 cycles of denaturation at 98°C for 20s, annealing at 60°C for 45 s, extension at 72°C for 30 s followed by stable extension at 72°C for 10 min and storage at 4°C (PCR instrument: ABI GeneAmp 9700 Thermo Fisher Scientific, USA). To ensure the absence of contamination and non‐specific amplification, PCR negative controls were performed in parallel with the environmental samples. In these reactions, the template DNA was replaced by an equivalent volume of molecular‐grade nuclease‐free water. All negative control reactions were subjected to the identical PCR thermal cycling conditions and downstream processing. The PCR products were recovered using a 2% agarose gel, purified using the DNA Gel Recovery and Purification Kit (PCR Clean‐Up Kit, China Yuhua Co. Ltd.), detected and quantified using Qubit 4.0 (Thermo Fisher Scientific, USA). The libraries were sequenced on the Illumina Nextseq2000 platform (commissioned by Shanghai Meiji Bio‐pharmaceutical Technology Co.).

### 2.4 Data Processing

Quality control of the double‐ended raw sequences employed fastq (Chen et al. 2018) (https://github.com/OpenGene/fastq v. 0.19.6) software and was spliced using FLASH (Magoč and Salzberg 2011) (http://www.cbcb.umd.edu/software/flash v. 1.2.11) software: Raw reads were processed to remove low‐quality sequences and potential sequencing errors by (1) quality filtering to trim reads based on mass value thresholds, (2) merging paired‐end reads to create longer sequences, (3) removing sequences with excessive mismatches in the overlapping region, and (4) demultiplexing samples based on barcode and primer sequences, while also correcting for potential orientation issues. Stringent parameters were applied to minimize errors during each step.

Using UPARSE v. 7.1 (Edgar 2013; Stackebrandt and Goebel 1994) software (http://drive5.com/uparse/), operational taxonomic unit (OTU) clustering was performed on the sequences after quality control splicing based on 97% similarity with chimeras excluded. Representative sequences and OTU tables obtained were identified by RDP Classifier (http://rdp.cme.msu.edu v.2.11) and compared to the 12S rRNA gene database (mitofish v. 3.75) for OTU species annotation, with a confidence threshold of 90%. The community composition of each sample was determined by calculating the relative abundance of taxa at phylum, class, order, family, genus, and species levels. Functional prediction analysis was conducted using PICRUSt2 (Douglas et al. 2020) (v. 2.2.0) software. Species identification was performed with reference to the MitoFish (http://mitofish.aori.u‐tokyo.ac.jp) and NCBI databases (http://www.ncbi.nlm.nih.gov), supplemented by consultation of published literature on the taxonomy of fish in the Yarlung Zangbo River (Xie et al. 2019; Zhang et al. 2022).

### 2.5 Statistical Analysis

To explore differences and similarities in fish community composition across habitat types/elevation categories, we conducted complementary non‐metric multidimensional scaling (NMDS) and analysis of similarity (ANOSIM). For the NMDS analysis, we used the Bray‐Curtis distance matrix derived from the OTU abundance data. Non‐metric multidimensional scaling was performed to downscale the high‐dimensional community data to a two‐dimensional space for visualization. The ordination was conducted with *k* = 2 dimensions, using 100 random starts to ensure a stable solution, and the stress value was calculated to evaluate the goodness‐of‐fit. In parallel, ANOSIM was performed using the same Bray‐Curtis distance matrix to statistically test for differences in fish community composition among the four elevation categories (E1–E4).

## 3 Results

### 3.1 Fish Species Composition

**FIGURE 2:**
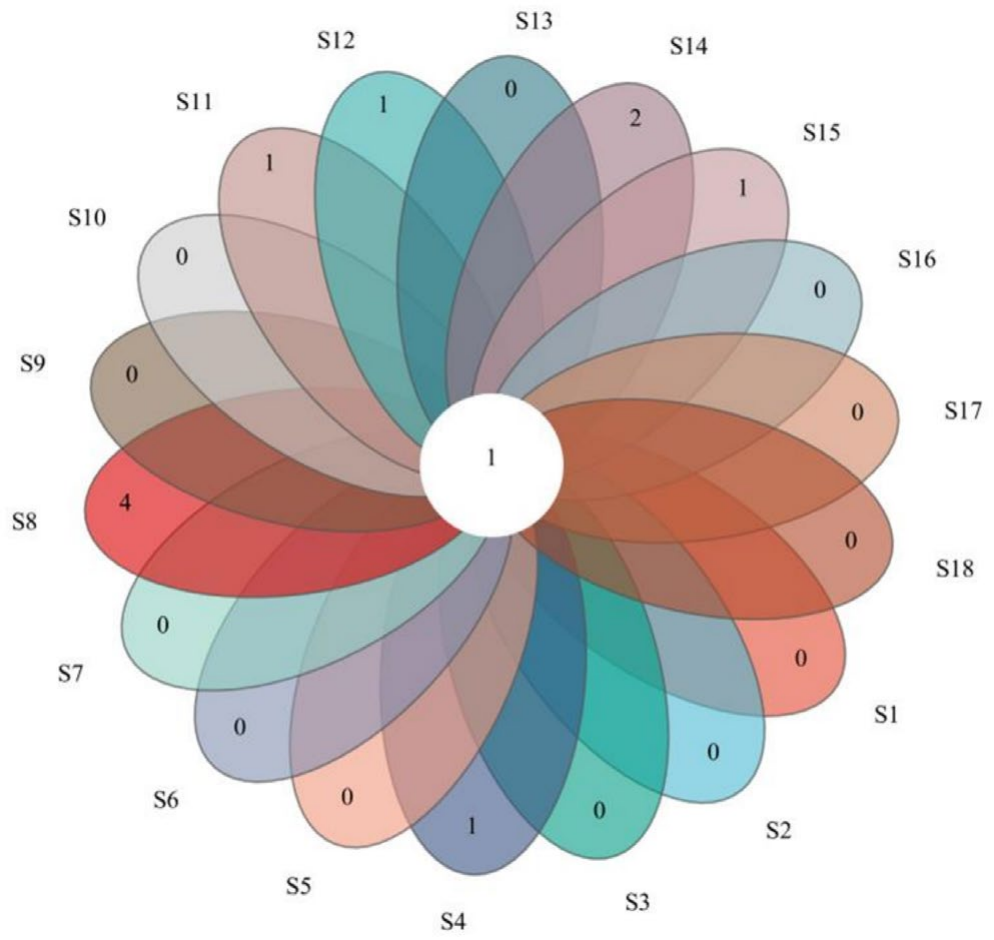
Venn diagram illustrates OTUs shared among sampling sites. The numbers in the overlapping segments represent the number of unique OTUs found in that combination of sites.

**FIGURE 3:**
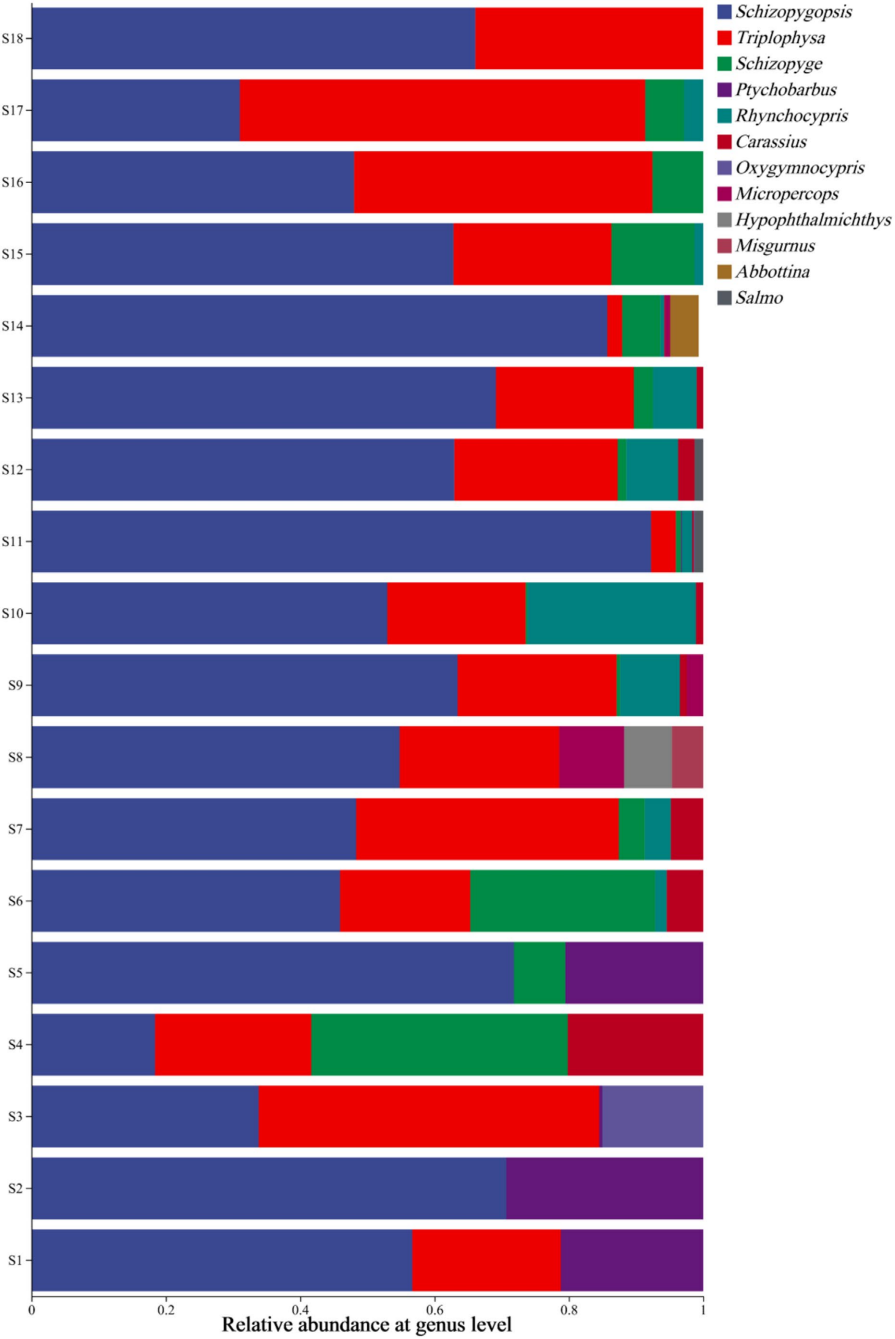
Relative abundance of fish genera at sampling sites.

Forty‐four fish OTUs were annotated to species at the 18 sampling sites comprising four orders, eight families, 13 genera, and 18 species based on sequence alignment and taxonomic classification. Consistent with our intention to prevent contamination, no amplification products were detected in any of the PCR negative controls. The genus *Rhynchocypris* was not identified to the species level (Table 1). Species included one state key protected fish, *Oxygymnocypris stewartii* . Six identified species are unique to Tibet, including *Schizopygopsis younghusbandi* , *Oxygymnocypris stewartii* , *Ptychobarbus dipogon* , *Schizothorax oconnori* , *Triplophysa tibetana* , and *Triplophysa stewarti* . Eight exotic species observed included *Misgurnus anguillicaudatus* , *Carassius auratus* , *Abbottina obtusirostris* , *Salmo trutta* , *Micropercops swinhonis* , *Pseudorasbora parva* , *Silurus asotus*, and *Hypophthalmichthys nobilis* . Cypriniformes dominated in species composition (16 species, 88.9%). A Venn diagram showed only a single OTU shared among all sampling sites but a low number of unique site‐specific OTUs (Figure 2), suggesting high similarity in community composition of sites. The genus *Schizopygopsis* ( *S. younghusbandi* ) was detected at all sampling sites with high relative abundance. The genus *Triplophysa* was also distributed at most sampling sites, with significant predominance at sites S3, S16, and S17 (Figure 3).

**TABLE 1:** Fish species detected at sampling sites in the Yarlung Zangpo River based on environmental DNA.

Read counts of species at each site																					
Order	Family	Genus	Species	S1	S2	S3	S4	S5	S6	S7	S8	S9	S10	S11	S12	S13	S14	S15	S16	S17	S18
Cypriniformes	Cobitidae	*Misgurnus*	*Misgurnus anguillicaudatus*	0	0	0	0	0	0	0	4467	0	0	0	0	0	0	0	0	0	0
	Cyprinidae	*Carassius*	*Carassius auratus*	0	0	0	19,359	0	5222	4641	74	982	1044	213	2346	940	114	0	0	0	0
		*Oxygymnocypris*	*Oxygymnocypris stewartii*	0	0	14,424	0	0	0	0	0	0	0	92	0	0	0	0	0	0	0
		*Ptychobarbus*	*Ptychobarbus dipogon*	20,342	28,106	441	0	19,705	0	0	0	0	0	119	0	0	0	0	0	0	0
		*Schizothorax*	*Schizothorax oconnori*	0	0	1	36,633	7333	26,386	3706	0	488	277	720	1217	2752	5478	11,846	7254	5547	0
		*Schizopygopsis*	*Schizopygopsis younghusbandi*	54,384	67,841	32,457	17,647	68,909	44,071	46,387	52,591	60,856	50,828	88,511	60,260	66,359	82,238	60,294	46,067	29,752	63,178
Cypriniformes	Gobionidae	*Abbottina*	*Abbottina obtusirostris*	0	0	0	0	0	0	0	0	0	0	0	0	0	4078	0	0	0	0
		*Pseudorasbora*	*Pseudorasbora parva*	0	0	0	0	0	0	0	0	0	0	0	0	0	639	0	0	0	0
	Leuciscidae	*Rhynchocypris*	*Rhynchocypris* sp.	0	0	0	0	0	1645	3724	0	8548	24,040	1488	7382	6221	524	1266	0	2739	0
	Nemacheilidae	*Triplophysa*	*Triplophysa brevicauda*	1	0	0	22,124	0	3704	10,116	1	0	41	0	1110	738	419	7650	7004	9964	10,753
			*Triplophysa dalaica*	0	0	0	0	0	0	148	1	0	602	0	899	432	0	0	0	179	28
			*Triplophysa orientalis*	0	0	33	0	0	0	1	30	14	5	1	73	1235	0	0	17	19	109
Cypriniformes	Nemacheilidae	*Triplophysa*	*Triplophysa scleroptera*	21,220	0	1	146	0	14,596	4527	544	20,804	18,202	3064	11,729	14,399	1713	14,879	28,227	2992	159
			*Triplophysa stewarti*	0	0	48,590	20	0	303	22,676	22,175	1902	908	470	9477	2867	1	7	7368	34,194	21,362
			*Triplophysa tibetana*	0	0	0	18	0	3	21	0	0	0	0	2	1	0	5	10	10,561	9
	Xenocyprididae	*Hypophthalmichthys*	*Hypophthalmichthys nobilis*	0	0	0	0	0	0	0	6837	0	0	0	0	3	0	0	0	0	0
Gobiiformes	Odontobutidae	*Micropercops*	*Micropercops swinhonis*	0	0	0	0	0	0	0	9227	2353	0	0	0	0	743	0	0	0	0
Salmoniformes	Salmonidae	*Salmo*	*Salmo trutta*	0	0	0	0	0	0	0	0	0	0	1269	1230	0	0	0	0	0	0
Siluriformes	Siluridae	*Silurus*	*Silurus asotus*	0	0	0	0	0	17	0	0	0	0	0	222	0	0	0	0	0	349

The number of species sequences was consistent with these findings, with *Schizopygopsis* distributed among all 18 sampling sites with a similar number of species, whereas the number of *Carassius auratus* , *Schizothorax oconnori* , and *Triplophysa stewarti* varied among sampling sites.

### 3.2 Fish Biodiversity

#### 3.2.1 Alpha Diversity Analysis

Alpha diversity analysis of the fish community was carried out by calculating the Chao1, Shannon, Simpson and ACE diversity indices and observed OTUs (*S*_obs_) (Table 2). The highest number of OTUs was in S12 (28), and the lowest was in S1 and S5 (8). The range of coverage was 0.9994–0.9999, indicating that the sequencing revealed virtually all OTUs, and reflects the precision of the samples. The range of Shannon's diversity index was 0.40038–1.85120, and Simpson's diversity index was 0.19093–0.85217. Shannon's index was highest at S17 and lowest at S11, whereas Simpson's index showed the reverse pattern, indicating that sampling site S17 showed higher community diversity and S11 lower community diversity. The ACE diversity index ranged from 8.385 to 33.056 and the Chao1 index was 8–30. The distribution trends of the two indices were similar, with the highest ACE and Chao1 indices observed at sites S18 and S14, respectively. The lowest ACE and Chao1 indices were found at site S5.

**TABLE 2:** Fish alpha diversity indices at sampling sites in the Yarlung Zangbo River.

Site	*S*_obs_	Shannon	Simpson	ACE	Chao1	Coverage
S1	8	0.98591	0.41498	8.582	8.00	0.99999
S2	9	0.60629	0.58555	11.295	9.75	0.99996
S3	11	1.02832	0.39312	32.185	17.00	0.99995
S4	19	1.52419	0.24662	22.200	21.00	0.99995
S5	8	0.76095	0.56361	8.385	8.00	0.99999
S6	22	1.38039	0.31430	29.362	23.50	0.99995
S7	24	1.60861	0.29251	26.100	25.00	0.99996
S8	22	1.41717	0.34589	25.908	24.00	0.99995
S9	14	1.08247	0.45819	15.382	14.50	0.99997
S10	16	1.15378	0.37918	17.593	16.30	0.99997
S11	20	0.40038	0.85217	24.498	25.00	0.99994
S12	28	1.31754	0.42483	29.394	28.60	0.99996
S13	24	1.11049	0.50645	28.339	29.00	0.99994
S14	20	0.63599	0.73981	24.393	30.00	0.99994
S15	20	1.11366	0.44011	21.690	21.50	0.99996
S16	18	1.31427	0.33344	19.411	18.30	0.99997
S17	20	1.85120	0.19093	22.222	20.50	0.99997
S18	21	1.01002	0.48178	33.056	24.00	0.99995

#### 3.2.2 Beta Diversity Analysis

**FIGURE 4:**
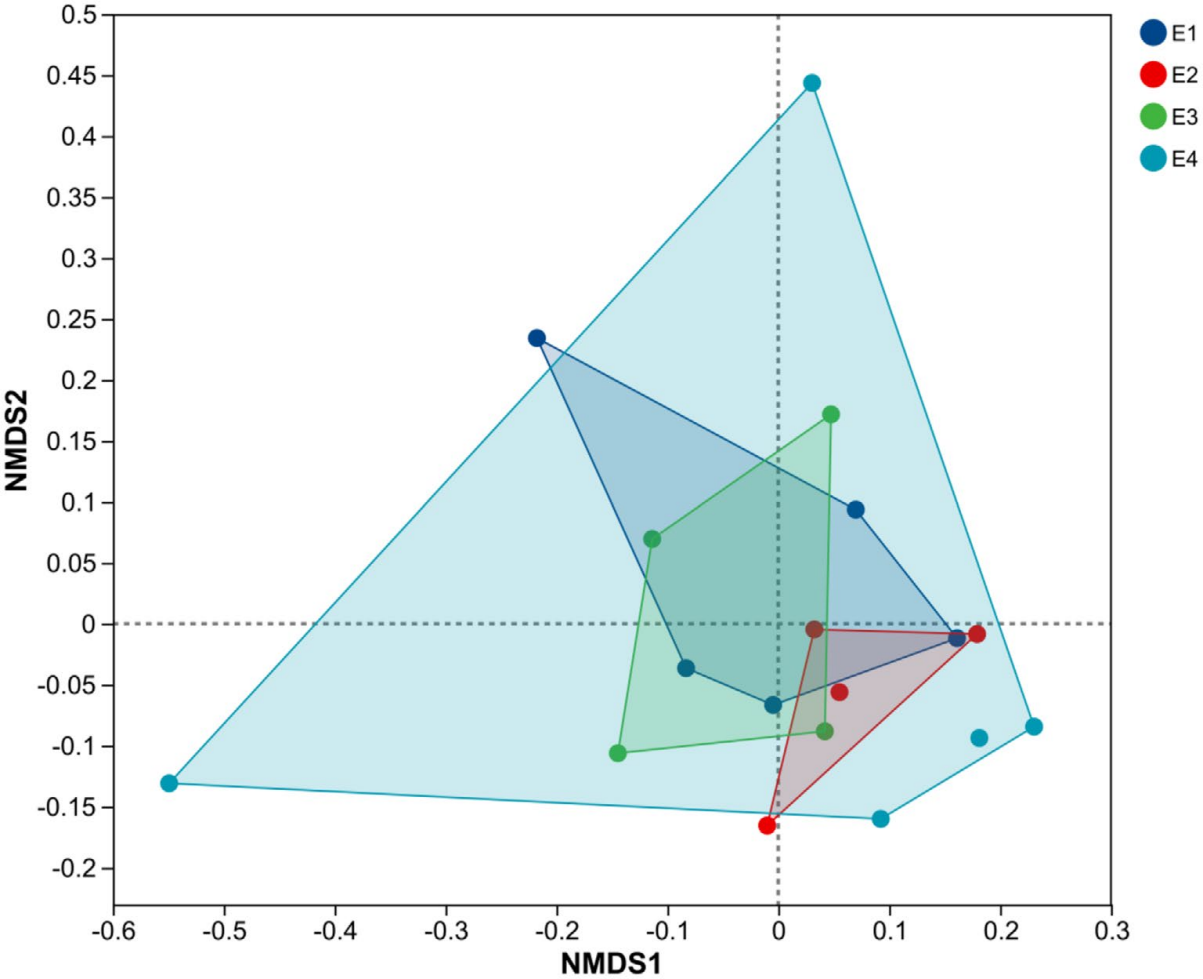
Non‐metric multidimensional scaling analysis (NMDS) plot based on Bray–Curtis dissimilarity. The colored symbols represent different elevation ranges: E1 (blue): 2931–3196 m (sampling sites S1, S2, S3, S4, S5); E2 (red): 3551–3600 m (sampling sites S6, S7, S8, S9); E3 (green): 3753–3996 m (sampling sites S10, S11, S12, S13); and E4 (cyan): 4470–4657 m (sampling sites S14, S15, S16, S17, S18).

Non‐metric multidimensional scaling analysis sequencing based on Bray–Curtis dissimilarity was used to illustrate the structure of fish communities at four elevation categories: E1 (2931–3196 m), E2 (3551–3600 m), E3 (3753–3996 m), and E4 (4470–4657 m) (Figure 4). The stress value of the NMDS plot was 0.109, indicating that the spatial relationships of the data were accurate. The E2 and E3 communities were in proximity in NMDS space and partially overlapped, suggesting fish community composition more similar than that of E1 to E4. The high elevation site E4 was shown by the NMDS to present a community composition distinct from the other sites. While it showed a degree of proximity to E2 and E3, indicating some shared characteristics, the overall clustering of E4 suggests a unique assemblage of species. Although the stress value suggests a good fit of the data, the low *R*‐value (*R* = 0.0878) and non‐significant *p*‐value (*p* = 0.145) obtained in the analysis of similarity (ANOSIM) indicate that the composition of fish communities does not differ significantly with elevation, implying overlap and gradual change rather than clearly delineated community groups.

### 3.3 Fish Community Diversity Relative to Environmental Factors

**FIGURE 5:**
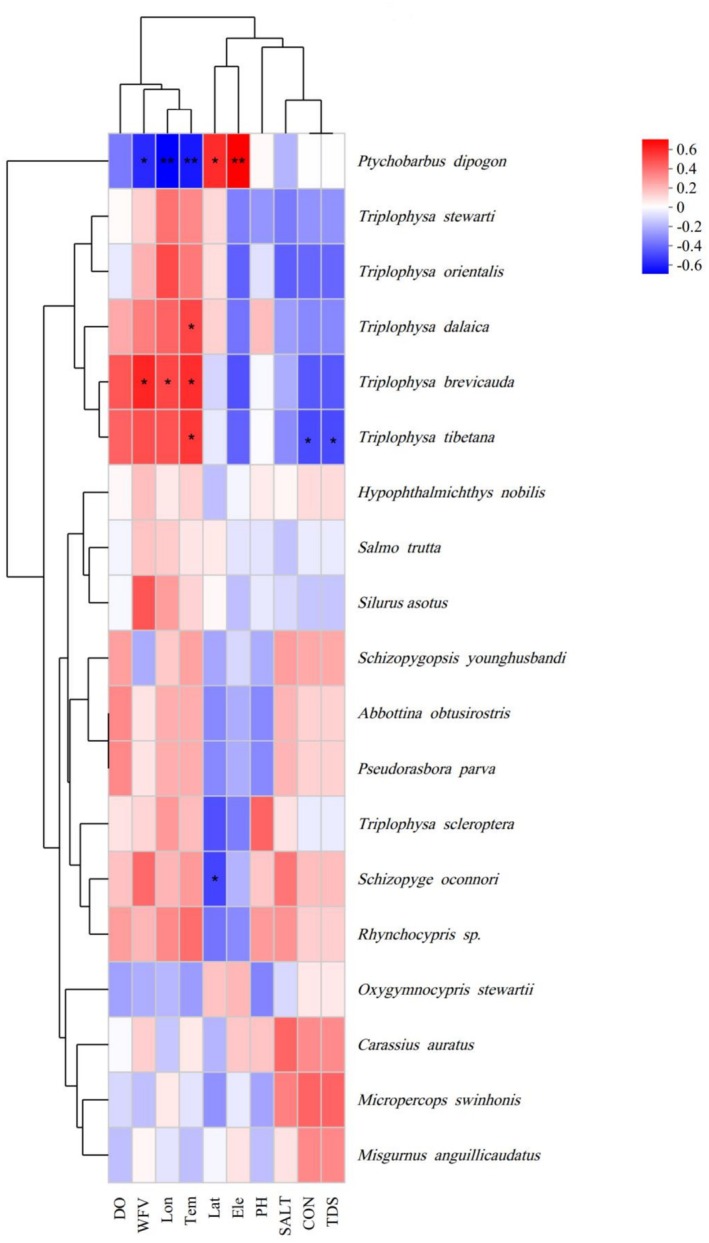
Correlation of fish species and environmental factors. Spearman's rank correlation coefficients are displayed, with color intensity indicating the strength and direction of the correlation (red = positive, blue = negative). Environmental factors include dissolved oxygen (DO), water flow velocity (WFV), longitude (Lon), water temperature (Tem), latitude (Lat), elevation (Ele), pH, salinity (SALT), conductivity (CON), and total dissolved solids (TDS). Significant correlations are marked with asterisks: *p < 0.05, **p < 0.01.

Spearman's rank correlation analysis revealed distinct associations between fish species presence and environmental variables including hydrological and geographic conditions (Figure 5). Occurrence of *Ptychobarbus dipogon* was significantly positively correlated with elevation (*p* = 0.003) and latitude (*p* = 0.021) and significantly negatively correlated with flow velocity (*p* = 0.019), longitude (*p* = 0.003), and water temperature (*p* = 0.010) (Figure 5).

## 4 Discussion

### 4.1 Environmental DNA Based Assay of Yarlung Zangbo River Fish Communities

We detected 18 fish species, including six endemic Tibetan fishes and one national grade 2 protected species, *Oxygymnocypris stewartii* . The number of species found was low compared to fish surveys based on traditional fishing methods, such as Gong et al. (2024), which reported 37 species, and Liu et al. (2021), which reported 35 species (Gong et al. 2024; Liu et al. 2021). This difference does not reflect absolute limitations of the eDNA method, but stems from the inherent limitations such as differences in the detectability of species due to variation in DNA shedding rates or primer biases during PCR amplification. *Salmo trutta*, and *Rhynchocypris* sp., not recorded in the study of Gong et al. (2024), were detected in this study, demonstrating the potential of eDNA technology to supplement results of traditional surveys Traditional methods benefit from the use of a combination of capture techniques (e.g., gillnets, ground cages, electrofishing), to enhance the completeness of findings. The eDNA method, despite the advantages of non‐invasiveness and high throughput, has limitations, since eDNA concentration is affected by dynamic processes including dilution, degradation, and diffusion in water, potentially resulting in failure to detect DNA of some low‐abundance species (Goldberg et al. 2016). *Schizopygopsis younghusbandi* was detected at all sampling sites with even distribution of sequence numbers, suggesting a relatively stable population despite potential fluctuations caused by seasonal changes or anthropogenic disturbances in the river system. The ecological success of *S. younghusbandi* in the Yarlung Zangbo River Basin is likely due to a combination of adaptations, including its ability to efficiently utilize benthic invertebrates as a food source, reproduce at low temperatures, and tolerate the chronically hypoxic conditions. These traits, working in concert, allow *S. younghusbandi* to thrive in the challenging alpine environment where other species struggle (Li et al. 2024).

The observed elevation pattern of *Schizothorax oconnori* and *Triplophysa stewarti* abundance may reflect physiological tolerance to colder, oxygen‐rich waters. Historical factors such as the post‐glacial expansion of suitable habitats, may have contributed to the current distribution patterns. This may indicate a lack of comprehensive sequence data for all *Rhynchocypris* sp. species in the NCBI database, or the presence of significant sequence similarity among closely related species, hindering accurate species‐level identification based on eDNA. This highlights the need for further taxonomic study of fish species in the basin (He et al. 2024). The differences in results of this study from those based on traditional fishing methods reflect limitations of both survey methods. The assessment of fish diversity in the Yarlung Zangbo River Basin may be improved by combining eDNA surveys with traditional capture and morphological identification with molecular analyses, to enhance understanding of species distribution and abundance.

### 4.2 Impact of Environmental Factors on Fish Communities in the Yarlung Zangbo River

Non‐metric multi‐dimensional scaling analyses did not show fish community composition to differ significantly with elevation (*R* = 0.0878, *p* = 0.145). Nonetheless, distribution patterns of communities at different elevations in the NMDS maps hint at an influence on fish community structure. The highest elevation community, E4, showed a clear separation from that of sites E1, E2, and E3, suggesting that elevation may influence community structure. This is consistent with the results of a previous study of fish diversity in high‐elevation rivers (Li et al. 2022). The ANOSIM analyses imply that there may be a continuum of change in fish community composition with elevation, rather than unique community patterns, possibly related to hydrological connectivity and migratory dispersal (Cui et al. 2024). Spearman correlation analyses revealed significant correlations of fish species presence with environmental factors. *Ptychobarbus dipogon* showed a significant positive correlation with elevation and a significant negative association with water temperature. *Triplophysa brevicauda* presence was significantly positively correlated with water flow velocity and temperature, indicating the sensitivity of this species to hydrological conditions. The positive correlation with longitude may reflect local/regional environmental factors that vary with east–west location, such as changes in river size or geological characteristics. *Triplophysa tibetana* occurrence was significantly positively correlated with water temperature and significantly negatively correlated with conductivity and total dissolved solids, which may indicate sensitivity to water quality. These results suggest the environmental factors that play a role in shaping the structure of the Yarlung Zangbo River fish community.

### 4.3 Exotic Fish Species in the Yarlung Zangbo River

Invasive alien species have become a serious threat to fish diversity in the Yarlung Zangbo River Basin (Ding et al. 2022). The presence of invasive species may change the community structure and impact the survival of native fishes through competition, predation, and disease transmission, reducing the stability and function of the ecosystem (Havel et al. 2015). Eight non‐native fish species were detected in this study: *Misgurnus anguillicaudatus* , *Carassius auratus* , *Abbottina obtusirostris, Micropercops swinhonis*, *Pseudorasbora parva* , *Salmo trutta, Silurus asotus*, *and Hypophthalmichthys nobilis*. *Misgurnus anguillicaudatus* and *C. auratus* exhibit broad tolerance and reproduction ability and may outcompete native species for resources. Beyond the general competitive threat posed by *Misgurnus anguillicaudatus* and *Carassius auratus* , the presence of predators such as *Salmo trutta* poses a direct risk to the recruitment of native species through possibly egg and juvenile predation. Furthermore, the filter‐feeding invasive *Hypophthalmichthys nobilis* competes directly with native planktivores for phytoplankton resources, potentially altering the base of the aquatic food web. The remaining detected exotic species, including *Pseudorasbora parva* and *Abbottina obtusirostris* , are characterized by their high adaptability and rapid reproductive cycles. These traits likely contribute to resource depletion and the displacement of less tolerant native taxa, thereby exacerbating the overall pressure on native ichthyofauna in the study area. eDNA technology has great potential for early detection of invasive species (Sepulveda et al. 2023) and monitoring the extent of their distribution, providing a basis for the development of management strategies (Rishan et al. 2023).

### 4.4 Factors Affecting eDNA Sampling and Interpretation

In a river system such as the Yarlung Zangbo, with high elevation, complex hydrological conditions, and unique ecology, the application of eDNA technology faces challenges that may affect the reliability and interpretation of data (Shen et al. 2024). The hydrological dynamics of the river, including strong seasonal runoff variation, rapid flow, and turbulent currents, may lead to uneven distribution and rapid downstream transport of DNA in the stream, potentially affecting the representativeness of sampling sites and the accuracy of species detection (Gao et al. 2023; Feng et al. 2023; Shu et al. 2020). Cold conditions in high‐elevation environments may slow the rate of DNA degradation, leading to the detection of DNA of dead or migrating organisms (Strickler et al. 2015). Despite the high sensitivity of eDNA technology, amplification bias and non‐specific binding of primer sets during PCR may lead to under‐ or over‐amplification of DNA of some species, affecting the assessment of species diversity and relative abundance (Fonseca 2018). The fish reference database used may be incomplete, especially of high‐elevation endemic species, resulting in inaccurate taxonomic identification or the omission of as‐yet undescribed cryptic species. This limitation was notably evident in our inability to achieve species‐level identification for the genus *Rhynchocypris*. Such challenges are frequently observed for endemic taxa in understudied biodiversity hotspots like the Tibetan Plateau. These database gaps can lead to underestimations of regional species richness and obscure the distribution ranges of endemic species, ultimately impacting the accuracy of conservation prioritization. This is a particular concern for Yarlung Zangbo River fish fauna, as the harsh environmental conditions can lead to rapid DNA degradation, making it difficult to amplify and identify certain species, which may have contributed to the relatively low number of fish species identified in this study. Finally, the geographic remoteness and complex topography of the Yarlung Zangbo River Basin increased the risk of potential contamination during sample collection and handling (Barnes et al. 2021) and may affect the accuracy of the results despite the stringent control measures taken. Nonetheless, the high sequence coverage values obtained (> 0.99994) indicate that the methodology used is robust and reflects the real situation of the fish communities in the region.

## 5 Conclusions

The environmental DNA metabarcoding technology applied to assess fish diversity in the Yarlung Zangbo River on the Tibetan Plateau overcomes the limitations of traditional survey methods in a high elevation habitat. Future exploration might include (1) longer time series eDNA data to track the dynamics of fish communities, especially in the context of increasing impacts of climate change and human activity; (2) integration of eDNA analyses with traditional fish survey methods for a more comprehensive assessment of biodiversity and to validate the accuracy and reliability of the eDNA technique; (3) in‐depth molecular ecology studies (i.e., studies that use molecular techniques to investigate ecological questions) to investigate the relationship of environmental factors with fish community structure in alpine ecosystems; and (4) quantitative PCR for eDNA studies targeting species or functional groups to provide assessment of population sizes to monitor endangered species and for early detection of invasive species. With continued development and improvement, eDNA technology will play an increasingly important role in biodiversity conservation, ecosystem management, and environmental monitoring and provide technical support for the sustainable development of global alpine ecosystems.

## Author Contributions

**Haiyu Wang:** data curation (equal), formal analysis (equal), methodology (equal), software (equal), validation (equal), visualization (equal), writing – original draft (equal), writing – review and editing (equal). **Qianqian Wu:** investigation (equal), methodology (equal), visualization (equal), writing – review and editing (equal). **Shenhui Li:** formal analysis (equal), investigation (equal), software (equal). **Hongyu Jin:** software (equal). **Zepeng Zhang:** data curation (equal), validation (equal). **Wanqiao Lu:** data curation (equal), formal analysis (equal). **Fei Liu:** data curation (equal). **Guishuang Wang:** visualization (equal). **Lei Li:** conceptualization (equal), funding acquisition (equal), investigation (equal), methodology (equal), project administration (equal), supervision (equal), writing – review and editing (equal).

## Conflicts of Interest

The authors declare no conflicts of interest.

## Data Availability

The raw sequencing data generated in this study have been deposited in the NCBI Sequence Read Archive (SRA) under the BioProject accessionnumber PRJNA1299613 (https://www.ncbi.nlm.nih.gov/bioproject/PRJNA1299613).
